# Supplementary material for: A Photoperiod-Regulating Gene CONSTANS Is Correlated to Lipid Biosynthesis in Chlamydomonas reinhardtii
Source: Biomed Res Int. 2015 Jan 15;2015:715020. doi: 10.1155/2015/715020 (PMC4310486; doi:10.1155/2015/715020)
Supplement: Supplementary file 1 — The standard curve of the biomass concentration and Nile red fluorescence value, and the gene primers for Real Time PCR. [file 715020.f1.pdf]

A photoperiod-regulating gene *CONSTANS* is correlated to lipid biosynthesis *in Chlamydomonas reinhardtii*

Xiaodong Deng1, Xinzao Fan1, Ping Li1, and Xiaowen Fei2*

1Key Laboratory of Tropical Crop Biotechnology, Ministry of Agriculture, Institute of Tropical Bioscience and Biotechnology, Chinese Academy of Tropical Agricultural Science, Haikou 571101, China.

2School of Science, Hainan Medical College, Haikou, 571101, China.

*Corresponding author. E-mail: Feixw2000@hotmail.com Tel: +86-898-66960173.
Fax: +86-898-66890978.

Abstract

Background

The regulation of lipid biosynthesis is essential in photosynthetic eukaryotic cells. Thus far, studies on lipid metabolism in microalgae have focused on the analysis of key enzymes and genes that are also involved in photosynthetic carbon flow. No regulatory genes have been reported in the lipid metabolism pathway. Plant *CONSTANS (CO)* gene regulates blooming by participating in photoperiod and biological clock. Apart from regulating photoperiod, the *Chlamydomonas CO* gene also regulates starch content by regulating the granule-bound starch synthase (GBSSI) gene. As such, the regulation of lipid biosynthesis is essential in photosynthetic eukaryotic cells.

Results

In this study, the detection of lipid content in HSM-S indicated that cells accumulated a significant amount of lipids at short-day conditions than at long-day conditions. While in *Chlamydomonas* CC425, the *CrCO* mRNA level decreased in the -S medium compared with that in the HSM medium which indicate that the *CrCO* mRNA level was negatively correlated to lipid accumulation at the -S conditions. The silencing of the *CrCO* gene via RNA interference (RNAi)

resulted in an increase in lipid content by 13.5% to 35.2%, which was analyzed via the Nile red fluorescence method, and an increase in triacylglyceride (TAG) level by 24.5%. *CrCO* RNAi strains accumulated more lipids at short-day conditions than at long-day conditions. The decrease in *CrCO* expression resulted in the increased expression of TAG biosynthesis-related genes, such as acyl-CoA:diacylglycerol acyltransferase, phosphatidate phosphatase, and phospholipid:diacylglycerol acyltransferase, whereas citrate synthase and fructose-1,6-bisphosphatase genes showed a decrease in their mRNA when the *CrCO* expression was suppressed. On the other hand, the overexpression of *CrCO* resulted in a decrease in lipid content by 26.2% to 36.0% and a decrease in TAG level by 19.4%. In summary, the results of this study revealed a relationship between *CrCO* gene and lipid metabolism in *Chlamydomonas*.

Conclusions

Observations made in this paper suggest that the regulation of the *CrCO* gene can control the lipid content of algae cells. The findings of this study suggest that increasing oil by suppressing *CrCO* expression in microalgae is feasible.

Keywords: *CrCO*; lipid biosynthesis; RNAi interference; *Chlamydomonas reinhardtii*; nutrition deprivation, photoperiodism

Introduction

Given the risk of depleting traditional fossil fuels such as oil and coal, people have now realized the urgent need to develop renewable energy sources. Thus, the use of biodiesel from microalgae as an important source of renewable energy and as a vital alternative energy source of future fossil fuel has attracted increasing attention from scholars and enterprises. Autotrophic microalgae convert solar energy into biomass energy, which fix a large amount of CO_2 and store the biomass energy as lipids in cells in specific strains. For a long time, studies on the lipid metabolism pathway of microalgae lagged behind studies on most crops such as rice, wheat, corn, and so on. More studies have been concentrated on the mechanism of lipid metabolism and high-density culture, which are significant to the genetic improvement of high quality strains and the industrialization of aquaculture.

Photoperiodism, a ubiquitous feature of plants, is the response of plants to the relative length of day and night. Photoperiodism is a key factor that affects plant conversion from vegetative growth to reproductive growth as well as the plant's flowering time, which is regulated by a large and complex genetic network [1]. *CONSTANS* (*CO*) is an important gene that regulates both plant photoperiod and flowering time [2, 3]. *CO* homologous genes were identified in a few plants, such as *Hd1* of rice [4], *TaHd1* of *Tritivum aestivum* [5], *StCO* of *Solanum tuberosum* [6,7], and *CrCO* of

Chlamydomonas reinhardtii [8] by the screening the library and by homology-based cloning with the *CO* gene sequence of *Arabidopsis*. Meanwhile, *CO* homologous genes have been cloned in different species, including rape plants, eastern cottonwood [9], barley [10], tomato [11], pharbitisnil [12], spiderflower [13], radish [14], pea [15], perennial ryegrass [16], and so on.

CO genes were found in multiple copies in most plant genomes that have been studied. A total of 17 homologous genes were found in *Arabidopsis* [17], 16 in rice [18], three in *Solanum lycopersicum* [19], four in *Brassica napus* [20], and two in *Picea abies* [21]. CO is a zinc finger transcription factor that contains B-box and CCT conservative domains and a variable region in the middle [22–25]. The N-terminal of CO has two sequential B-box domains designated as double B-box (DBB). The B-box domain of CO interacts with proteins [26], in which the cysteine and histidine that bind to zinc ion are highly conserved. Conservative amino acid mutations resulted in a delay in flower production [27]. Ben-Naim used the B-box of TCOL1 as bait to analyze its interaction with immunophilin and/or other proteins that contain B-box and determined that B-box mainly functions with proteins [28]. The C-terminal of the CO protein is composed of 70 to 80 amino acids, in which approximately 40 are highly conserved in the CO family [27, 29]. Moreover, the CCT domain of CO contains a nuclear localization signal that interacts with COP1 of the ubiquitin ligase [30]. In the present study, the mechanism of combining CO with DNA is unclear, although speculation indicated that CO binds to DNA via its CCT domain to form a complex compound.

Aside from the DBB zinc finger and the CCT domain, the B-box family of *Arabidopsis* also contains DBB homologous CO subfamilies, which have two DBB domains in the N-terminal that are separated by 8 to 15 amino acids, whereas the C-terminal does not contain any CCT domains. DBB homologous subfamilies are encoded by eight genes, namely, *DBB1a* (At2g21320), *DBB1b* (At4g38960), *DBB2* (At4g39070), *DBB3* (At1g78600), *DBB4* (At4g10240), *STO* (At1g06040), *STH* (At2g31380), and *STH2* (At1g75540) [31]. To date, four of the eight genes have been found to be involved in light-mediated plant growth and development. *STO* has a function in plant salt tolerance and negatively regulates phytochrome and blue light signal transduction pathway [32]. *STH* have a similar function with *STO*, and they both interact with ubiquitin ligase *COP1* [33]. Both *DBB3* and *STH2* participate in regulating plant hypocotyl elongation, early chloroplast development, anthocyanin accumulation, and in the positive regulation of de-etiolation in *Arabidopsis* [33–35].

Functions of CO in microalgae have also been reported. Serrano et al. determined that CrCO regulates light cycle in *Chlamydomonas*, and both knockdown and overexpression of *CrCO* changed the diurnal cycle of the cells. Thus, related gene expression and physiological functions are regulated [8]. Ral et al.

determined that CrCO participates in starch synthesis by regulating the *GBSSI* gene [36]. *Chlamydonomas CO* gene reported by Serrano et al. was used to perform a BLASTP search in the *Chlamydonomas* database Phytozome, and only one homology, g6302, was found (100% identified). Other homologous protein containing the B-box domain was not detected. Nevertheless, seven genes that encode proteins containing the CCT domain were homologous to the *CrCO* gene. Based on the number and conservative property of B-box, 17 genes of the *CO* gene family can be divided into three subgroups in *Arabidopsis* [27, 29]. In addition, eight genes encoded proteins that are homologous to the DBB protein subfamily, namely, DBB1 to DBB4, STO, STH, and STH2 [31]. These genes regulate multiple physiological functions in plants, such as bloom, biological clock, photoperiod, growth and development (DBB1 to DBB4), salt-tolerance (STO), light signal transduction, hypocotyl elongation (DBB3 and STH2), early formation of the chloroplast, and accumulation of anthocyanin [32–35]. Compared with the mass of genes encoding the DBB subfamily of the CO protein in Arabidopsis, only one gene encoding the CO protein was found in *Chlamydomonas*, which indicates that higher plants and single CO gene in *Chlamydomonas* had complex functions such as the regulation of flower production and multicellular development.

To date, CrCO have not been proven to be involved in regulating lipid metabolism. In the field of microalgae lipid metabolism mechanism research, studies have concentrated on genes involved in the lipid synthesis pathway and photosynthetic carbon metabolism pathway, not on regulating genes. The present study discussed the function of *CrCO* gene in lipid accumulation in microalgae cells via the knockdown and overexpression of *CrCO* in *Chlamydomonas*. Furthermore, the relationship between *CrCO* gene and lipid metabolism was revealed by analyzing lipid accumulation at adverse -P and -S conditions and at long-day and short-day conditions.

Results and analysis

1. *Chlamydomonas* accumulated more lipids in short day (SD) than in long day (LD) under –S conditions .

C. reinhardtii CC425 was inoculated in a 50 mL Erlenmeyer flask containing an HSM medium and grown until the mid-log phase (2x10e^{6}). The cells were collected after centrifugation and then equally divided into three parts. Each triplet was inoculated in 30 mL medium of HSM, HSM-S, and HSM-P and grown at LD condition (16 h light and 8 h dark). Identical triplets were grown in SD conditions (8 h light and 16 h dark). The cells grew slightly slower in SD conditions than in LD conditions in HSM. Moreover, cell proliferation was greatly reduced in HSM-S compared with

that in HSM in LD or SD conditions, whereas the length of daylight did not affect cell growth in HSM-P (Figures 1 and 2). The lipid contents in both HSM and HSM-P in SD conditions were no significant difference compared to the corresponding lipid contents in LD conditions (Figures 1 and 2), whereas the lipid content significantly increased in SD conditions compared with that in LD conditions in HSM-S.

2. Analysis of the relationship between *CrCO* mRNA level and lipid accumulation at -S and -P conditions.

The *CrCO* mRNA level remarkably decreased in HSM-P compared with that in HSM. Moreover, the *CrCO* mRNA level decreased in HSM-S compared with that in the control sample (Figure 3). The lipid content remarkably increased in HSM-S compared with that in the control sample (Figure 1). The results illustrate that the *CrCO* mRNA level was negatively correlated with lipid accumulation at -S conditions.

3. Silencing of *CrCO* gene increases TAG content in *C. reinhardtii*

Approximately 1230 bp full-length CDS of *CrCO* DNA fragment was amplified via PCR and cloned into pMD18T and sequenced thereafter. This fragment exhibited 100% homology with the *Chlamydomonas CO* gene (g6302.t1). To determine the relationship between *CrCO* expression and lipid accumulation, the effects of the artificial silencing of *CrCO* gene on the lipid content of *C. reinhardtii* were examined. Based on the *CrCO* (g6302.t1) sequences of the gene retrieved from the Phytozome *C. reinhardtii* database (http://www.phytozome.net/), primers used to amplify the fragment of the coding region of *CrCO* were designed. The DNA fragments were subcloned and then used to generate *CrCO* RNAi constructs pMaa7IR/CrCO IR. More than 100 positive transformants were obtained after transforming the silencing construct into *C. reinhardtii* CC425. Three transgenic algae were selected to measure the lipid content and mRNA levels of the target gene. Strains transformed with the vector pMaa7IR/XIR were used as control samples. In cells harboring the *CrCO* construct, analysis results of the transgenic lines via the Nile red fluorescence method indicate an increase in the lipid content by 13.5% to 35.2% (Figure 4B) after 10 days of cultivation. The TAG level of the transgenic strain CrCO RNAi18 increased by 24.5% compared with that in the control sample (Figure 4C). To evaluate the effectiveness of the RNAi construct, the abundance of the target gene-specific mRNA in transgenic algae was analyzed via real-time PCR. The *CrCO* mRNA abundance decreased by 90.4% to 95.2% (Figure 4D), which indicates the high-efficiency silencing by these constructs.

Subsequently, the mRNA levels of phospholipid:diacylglycerol acyltransferase *PDAT3,* acyl-CoA:diacylglycerol

acyltransferase *(DGAT2*), phosphofructokinase *(PFK2*), fructose-1,6-bisphosphatase *(FBP1),* citrate synthase *(CIS*), and phosphatidate phosphatase *(PAP2)* genes were measured in transgenic strain CrCO RNAi18. Genes such as *PDAT3, DGAT2, and PAP2* are directly related to lipid synthesis, which increase the mRNA levels in transgenic strain compared with that in non-transgenic *C. reinhardtii* CC425 and Maa7IR/XIR transgenic algae. On the other hand, the *CIS* gene, a key enzyme in tricarboxylic acid cycle, and the *FBP1* gene, which is found in gluconeogenesis, showed a decrease in their mRNA in the transgenic strain (Figure 5). The results indicate the regulation of *CIS* and *FBP1* genes by *CrCO*, which exhibits negative effects on the regulation of the expression of lipid biosynthesis genes, such as *PDAT3, DGAT2, and PAP2*. The *FBP1* and *CIS* genes exhibited decreased mRNA in the transgenic strain, CrCO RNAi18, and a glycolysis enzyme, PFK2, exhibited increased mRNA in the transgenic strain, which indicate that more carbon are introduced to the fatty acid and lipid synthesis (Figure 5).

Results similar to the above discussion were obtained via Nile Red staining. More oil droplets were found in CrCO RNAi18 transgenic algae compared with that in pMaa7IR/XIR transgenic algae, as determined via microscopic analysis (Figure 6). This result indicates an increase in cell lipid content via the regulation of *CrCO* gene expression.

Considering that *CrCO* gene regulates photoperiod, would the lipid content of cells at SD or LD conditions change via RNAi-initiated *CrCO* knockdown? The results indicate that transgenic strains exhibited higher lipid content at SD condition than at LD condition (Figures 7A and 7B). The lipid content was measured at both -S and -P conditions in transgenic strains, and the results indicate that more lipids were accumulated at SD condition than at LD condition (Figures 7C–7F). Thus, more lipids are produced in RNAi transgenic strains compared with that in the control sample regardless of the cultivation conditions.

4. Overexpression of *CrCO* reduced the lipid content of *C. reinhardtii*

The increase in lipid content caused by the RNAi silencing of *CrCO* suggests the effect of the expression of these genes on the biosynthesis of triglycerides in *C. reinhardtii.* Thus, the capacity of *CrCO* overexpression to reduce the lipid content of C. reinhardtii was determined. Vector pCAMCO, which expresses *CrCO* gene from the CAMV 35S promoter, was introduced into C. reinhardtii. The lipid contents and growth rate of three randomly selected transgenic algae were determined in each transgenic algae line. The overexpression of the *CrCO* gene increased the growth rate of the algae in the early stages from day two to day four (Figure 8A). Moreover, the overexpression of *CrCO* decreased the lipid content of the transgenic algae compared with that in the control pCAMBIA1302 transgenic algae lines. For

example, six days after the growth of algae in the HSM medium in full daylight, the lipid contents of *CrCO*-overexpressing transgenic lines decreased by 26.2% to 36.0%, as determined via the Nile Red fluorescence method (Figure 8B). The TAG level of the transgenic strain, pCACO64, decreased by 19.4% compared with the TAG level of the control sample (Figure 8C). Compared with the mRNA levels of pCAMBIA1302 transgenic strains, the mRNA levels of *CrCO* increased by 27 to 29 times (Figure 8D). In summary, the overexpression of *CrCO* gene decreases lipid synthesis in cells. Decreased lipid content was also observed via Nile red dye staining (Figure 9). Fewer oil droplets were found in *CrCO*-overexpressed transgenic algae compared with the that in the control sample.

Discussion

Chlamydomonas CO has a typical DBB zinc finger domain and a CCT domain in the C-terminal, which has been proven to regulate photoperiod. Moreover, *CrCO* has a function in controlling starch content in *Chlamydomonas* by regulating the effects of *GBSSI* gene expression. In this study, *CrCO* was shown to regulate lipid accumulation. The mRNA level of the CO gene of *Chlamydomonas* decreased at -S condition via digital gene expression (DGE) profiling. Considering the increase in the lipid content at -S condition in cells, knockdown and overexpression of the *CO* gene in *Chlamydomonas* cells were performed to determine the relationship between the *CO* gene and lipid accumulation. The results indicate that the *CO* gene was closely correlated to lipid accumulation because the silencing of the *CO* gene results in an increase in the lipid content and the overexpression of the *CO* gene results in a decrease in the lipid content. In addition, the silencing of the *CO* gene caused the mRNA level of genes to contribute to lipid synthesis, such as *DGAT2*, *DGAT1*, and *PAP2*, which were optimized at daytime. Therefore, we hypothesized that *CrCO* facilitates indirect lipid production by regulating gene-encoding enzymes in the lipid synthesis pathway, namely, DGAT1, DGAT2, and PAP2. The SD condition exhibited positive effects on lipid accumulation compared with the LD condition, which was more significant in the -S condition. This conclusion was consistent with the findings that lipid content changes in *CO* knockdown transgenic algae strains. In future studies, emphasis must be given on how the *CO* gene regulates lipid synthesis gene expression, such as those of *DGAT1*, *DGAT2*, and *PAP2*.

Materials and methods

Bioinformatics, algal strain, cultivation conditions, and biomass assay

The information on the *Chlamydomonas CO* gene (g6302) was obtained from the Phytozome V9.1 *Chlamydomonas* database (http://www.phytozome.net). *C. reinhardtii* CC425 (mt) was purchased from

the *Chlamydomonas* Genetics Center at Duke University. The cells grown on tris-acetate-phosphate (TAP) agar plate were inoculated into 100 mL Erlenmeyer flasks containing 50 mL of HSM, P-deficient HSM (HSM-P), and S-deficient HSM (HSM-S) media [37]. The HSM medium was composed of NH_4Cl (0.500 $g \cdot L^{-1}$), MgSO4·7H_2O (0.020 $g \cdot L^{-1}$), $CaCl_2 \cdot 2H_2O$ (0.010 $g \cdot L^{-1}$), K_2HPO_4 (1.440 $g \cdot L^{-1}$), KH_2PO_4 (0.720 $g \cdot L^{-1}$), NaAc (2.000 $g \cdot L^{-1}$), H_3BO_3 (0.001 $g \cdot L^{-1}$), $MnCl_2 \cdot 4H_2O$ (0.005 $g \cdot L^{-1}$), $ZnSO_4 \cdot 7H_2O$ (0.022 $g \cdot L^{-1}$), $FeSO_4 \cdot 7H_2O$ (0.005 $g \cdot L^{-1}$), $CoCl_2 \cdot 6H_2O$ (0.002 $g \cdot L^{-1}$), $Na_2Mo_7O_{24} \cdot 4H_2O$ (0.002 $g \cdot L^{-1}$), and $Na_2 \cdot$EDTA (0.050 $g \cdot L^{-1}$). The HSM-P medium contained K_2HPO_4 and KH_2PO_4, which can be replaced with KCl. The HSM-S medium contained $MgSO_4 \cdot 7H_2O$, which can be replaced with $MgCl_2.6H_2O$. Generally, cultures were maintained in an incubator shaker at a rate of 230 rpm at 25 °C and then exposed to continuous illumination at a light intensity of 150 $\mu mol \cdot m^{-2} \cdot s^{-1}$. The samples tested in LD conditions were incubated in a light time of 16 h at daytime and 8 h at dark, whereas samples tested in SD conditions were incubated in a light time of 8 h at day time and 16 h at dark.

Biomass concentration (g/L) was determined by measuring the optical density of the samples at 490 nm (OD490), as described by an earlier study [38]. To generate the standard curve, a series of C. reinhardtii CC425 samples of different biomass concentrations was collected. The OD490 value and cell dry weight were gravimetrically determined using dried cells to plot the standard curve of OD490 versus biomass concentration (g/L). The samples were diluted to appropriate ratios to ensure that the measured OD490 values ranged from 0.15 to 0.75, if applicable. The biomass concentration was then calculated using the following formula: cell dry weight (g/L) = 0.7444*OD490-0.0132 (Supplementary data Figure 1).

Lipid content analysis

Nile Red fluorescence method and GC/MS were performed to determine lipid and TAG levels [39,40]. The algal cells were directly stained with 0.1 mg/mL Nile Red for 10 min, and fluorescence was then measured at excitation and emission wavelengths of 470 and 570 nm, respectively. The fluorescence value was calculated using the equation: FD (470/570)= (A2-A1), where A2 is the fluorescence value of the algal cells after staining with Nile Red, and A1 is the fluorescence of algal cells before staining

(Supplementary data Figure 2). Total lipid extraction was carried out according to a modified method. Logarithmic-phase algal cells were collected after centrifugation and extracted using an extraction buffer (methanol:chloroform:methanoic acid, 2:1:0.1), 1 M KCl, and 0.2 M H_3PO_4. The lipids were obtained after centrifugation at 13780 ×*g* for 3 min. For TAG separation, Si60 silica TLC plates for thin-layer chromatography (TLC) was used. The TLC plates were immersed in 0.15 M $(NH4)_2SO_4$ for 30 s and stored in an airtight container for two days. The plates were then placed in an oven at 120 °C for 2.5 h and cooled at room temperature. The samples were then added with N_2 flow. TAGs were observed on TLC plates via iodine staining. Lipid analysis was conducted as previously described. Fatty acid methyl esters derived from TAG were analyzed via GC/MS [41]. For microscopic assay, images were acquired using a Nikon 80i Fluorescence Microscope after the cells were stained with Nile Red. Nile Red signals were captured at an excitation wavelength of 480 nm, and emission was obtained between 560 and 600 nm [42–44]. A total of 30 cell lipid droplets from each algal strain were examined to determine the difference between the lipid contents.

RNA extraction

Total RNA was prepared as described by Li et al. with modification [45]. Cells from 10 mL of cultivated algae were collected after centrifugation at 10000 ×*g* for 1 min. After a series of extractions using phenol–chloroform, nucleic acids were precipitated with two volumes of absolute ethanol and then washed with 75% ethanol. Finally, the air-dried pellet was dissolved in 40 μL of RNase-free water. RNA concentration was determined via spectrophotometry, and the integrity was examined via agarose gel electrophoresis.

Cloning of the *CrCO* gene

The first strand of cDNA was synthesized using SuperScriptTM III Reverse Transcriptase (Invitrogen, USA) according to the manufacturer's instructions. A fragment of the *CrCO* gene was amplified via

polymerase chain reaction (PCR) by using primers CrCOL: 5'-ATGTCGAGTTGCGTCGTGTGCG-3' and CrCOR: 5'- TTAGCACTCAGCGTCCAGGACCTCG-3'. PCR reactions were performed in a final volume of 25 μL containing 1× PCR reaction buffer, 2 mM $MgCl_2$, 0.4 μmol of each primer, 0.25 mM dNTPs, 1 μL of DMSO, 0.5 M Betain, and 0.5 U Taq DNA polymerase (Promega, USA) according to the following program: 4 min at 95 °C; 35 cycles of denaturation for 40 s at 95 °C, annealing for 40 s at 58 °C, and elongation for 20 s at 72 °C; 10 min at 72 °C. After purification using the EZ-10 Spin Column DNA Gel Extraction Kit (BBI, Canada), the DNA was inserted into vector pMD18-T following the manufacturer's instructions (TaKaRa, Japan). The resulting plasmid was designated as pMD18T-CrCO. The sequences of the cloned *CrCO* gene were verified via double-stranded sequence analysis (Shanghai Sangon Biological Engineering Technology & Services Co., Ltd).

Construction of the RNAi vector against the *CrCO* gene

A fragment of *C. reinhardtii* 18S gene was amplified with primers 5'-CGAACTTCTGCGAAAGCAT-3' and 5'- TCAGCCTTGCGACCATACT-3' and then inserted into pMD18-T to produce pMD18T-18S. The fragment of *CrCO* and its reverse complementary sequences were amplified via PCR by using pMD18T-CrCO as a template and the following primers: CrCORNAiL: 5'- AGCTGCTACGCACGAGACCG -3', CrCORNAiR: 5'- GCCCATGTCGAGCCAGTTGT -3. The PCR fragment was then digested with KpnI/BamHI and HindIII/SalI and was inserted into the corresponding cloning sites of pMD18T-18S to yield pMD18-CrCOF-18S-CrCOR, which contained an inverted repeat sequence of *CrCO* (CrCO IR). pMD18-CrCOF-18S-CrCOR was double-digested with KpnI and HindIII to obtain CrCO IR. Finally, the CrCO IR was inserted as a blunt-end fragment into EcoRI-digested pMaa7/XIR to yield pMaa7IR/ CrCO IR.

Construction of overexpression vector of *CrCO* gene for *Chlamydomonas*

To construct the overexpression vector of the *CrCO* gene, the coding sequence of *CrCO* was amplified via PCR by using pMD18T-CrCO as a template and primers 5'-AAAGATCTAATGTCGAGTTGCGTCGTGTG-3' and 5'-AAACTAGTTTAGCACTCAGCGTCCAGGA-3'. The fragment was digested with NcoI/SpeI and inserted into similarly digested pCAMBIA1302 to produce pCAMCO, which allows the overexpression of *CrCO*.

Transformation of *Chlamydomonas*

The transformation of *C. reinhardtii* strain CC425 was performed as described by Kindle [46]. *C. reinhardtii* cells were grown in a TAP medium to a cell density of (1 to 2) × 10^6 cells/mL. The cells were collected after centrifugation, washed twice, and resuspended in the TAP medium to a cell density of approximately 1 × 10^8 cells/mL. Plasmid DNA was introduced into the cells via the glass bead procedure. In each case, 2 μg of plasmid DNA was included in a mixture containing 400 μL of cells, 100 μL of 20% polyethylene glycol, and 300 mg of sterile glass beads. The reaction was mixed for 15 s on a benchtop vortex. To allow the induction of RNAi or gene expression, the cells were allowed to recover for 1 d before plating onto selective media. RNAi transformants were selected on the TAP medium containing 1.5 mM L-tryptophan, 5 μg/mL paromomycin, and 5 μM 5-FI. pCAMCO transformants were selected on the TAP medium containing 50 μg/mL hygromycin. The plates were incubated under dim light (approximately 50 $\mu mol \cdot m^{-2} \cdot sec^{-1}$ photosynthetically active radiation). The isolated transgenic strains were kept at a constant selective pressure.

Quantitative real-time PCR

The samples were subjected to real-time PCR analysis as described by Fei et al. [47]. RNA was extracted using a TRIzol Reagent (Shanghai Sangon Biological Engineering Technology & Service Co.). Single-strand cDNA was synthesized using a Invitrogen SuperScriptTM III cDNA synthesis kit with 100 ng of RNA and random primers. The synthesis was performed at 65 °C for 5 min, 25 °C for 5 min, and 42 °C for 50 min. Real-time PCR was performed using a BioRad iCycler iQ Real-Time PCR Detection System with SYBR Green as the fluorescent dye. Each reaction was performed in a final volume of 25 μL with the following components: 0.2 pmoles of each primer, 1 μL of cDNA, 12.5 μL of SYBR Green Mix (Invitrogen SYBR GreenER qPCR), and water to adjust the volume to 25 μL. The iCycler protocol was as follows: denaturing at 95 °C, 5 min; 40 cycles of (denaturing at 95 °C, 30 s; annealing at 54 °C, 30 s; amplification at 72 °C, 15 s). The specificity of the PCR amplification was examined using a melting curve program (55 °C to 100 °C at a heating rate of 0.5 °C/s). 18S rRNA was used as the control sample with primers 18SrRNAF (5'-TCAACTTTCGATGGTAGGATAGTG-3') and 18SrRNAR (5'-CCGTGTCAGGATTGGGTAATTT-3'). 18S rRNA expression was measured and determined to be constant at all conditions. The gene-specific primers listed in Supplementary data table 1 were used to evaluate the quantity of target cDNA. The amplification rate of each transcript (Ct) was calculated via the

PCR baseline-subtracted method and performed in the iCycler software at a constant fluorescence level. Cts were determined over three repeats. Relative fold differences were calculated based on the relative quantification analytical method (2-ΔΔCT) by using 18s rRNA amplification as the internal standard [48].

Authors' contributions

XDD performed the molecular biology studies, participated in data analysis, and drafted the manuscript. PL performed the detection of the mRNA levels of the genes and documented all microscopic images. XZF performed the lipid and TAG content detection. XWF performed the statistical analysis, conceived the study, participated in designing the study, coordinated the research, and assisted in drafting the manuscript.

Ethics statement

This paper does not contain any studies with human or animal subjects performed by other authors.

Acknowledgement

This study was supported by the National Natural Science Foundation of China (31160050, 31360051, 31000117), Major Technology Project of Hainan (ZDZX2013023-1), National Nonprofit Institute Research Grants (CATAS-ITBB110507, 130305), Funds of Hainan Engineering and Technological Research (GCZX2011006, GCZX2012004, GCZX2013004), and Natural Science Foundation of Hainan Provence (313077).

References

1. Russell WK, Stuber C(1983) Effects of photoperiod and temperatures on the duration of vegetative in maize. Agro J 75:795-802
2. Imaizumi T, Schultz TF, Harmon FG, et al. (2005) FKF1, F-box protein mediates cyclic degradation of a repressor of CONSTANS in Arabidopsis. Science. 309:293-297.
3. Kobayashi Y, Weigel D. (2007) Move on up, it's time for change-mobile signals controlling photoperiod-dependent flowering. Genes Dev 21: 2371-2384.
4. Yano M, Katayose Y, Ashikari M, et al. (2000) Hd1,a major photoperiod sensitivity quantitative trait locus in rice,is closely related to the Arabidopsis flowering time gene CONSTANS . Plant Cell 1212: 2473-2484.
5. Nemoto Y, Kisaka M, Fuse T, et al.(2003) Characterization and functional analysis of three wheat genes with

homology to the CONSTANS flowering time gene in transgenic rice. Plant J 361: 82-93.

6. Drobyazina PE, Khavkin EE. (2006) A Structural homolog of CONSTANS in potato. Plant Physiol 53:698-701.
7. Polina E, Drobyazina, Emil E, et al. (2011) The structure of two CONSTANS-LIKE1 genes in potato and its wild relatives. Gene 471: 37-44.
8. Serrano G, Herrera-Palau R, Romero JM, et al.(2009) Chlamydomonas CONSTANS and the evolution of plant photoperiodic signaling. Curr Biol 195: 359-368.
9. Yuceer C, Harkess RI. (2002)Structure and developmental regulation of CONSTANS-LIKE genes isolated from Populus deltoids. Plant Sci 163:615-625.
10. Griffiths S, Dunfordr P, Coupiand G. (2003) Laurieda, The evolution of CONSTANS-LIKE gene families in barley, rice and Arabidopsis. Plant Physiol 131:1855-1867.
11. Bennaimo, Eshed R, Parni S A, et al. (2006) The CCAAT binding factor can mediate interactions between CONSTANS-LIKE proteins and DNA. Plant J 46:462-476.
12. Iauj, Yu J, Mcintosh I, Kende, Zeevaart JA (2001) Isolation of a CONSTANS ortholog from Pharbitis nil and its role in flowering. Plant Physiol 125: 1821-1830.
13. Schranz M E, Mitcheio T (2006) Independent ancient polyploidy events in the sister families Brassicaceae and Cleomaceae. Plant Cell 18:l152-1165.
14. Chae S, Jung J Y, An G. Expressed sequence tags of radish flower buds and characterization of a CONSTANS LIKE 1 gene. Mol Cells[J].1998, 8(4):452-458.
15. Hecht V, Foucher F, Ferrandiz C, Macknight R (2005) Conservation of Arabidopsis flowering genes in model legumes. Plant Physio1 137:1420-1434.
16. Martin J, Strgaard M, Andersen CH, Nieisen KK (2004) Photoperiodic regulation of flowering in perennial ryegrass involving a CONSTANS-LIKE homolog. Plant Mol Biol 56:159-169.
17. Robson F, Costa MM, Hepworth SR, Vizir I (2001) Functional importance of conserved domains in the flowering- time gene CONSTANS demonstrated by analysis of mutant alleles and transgenic plants. Plant J 28:619-631.
18. Griffiths S, Dunfordr P, Laurieda G (2003)The evolution of CONSTANS-LIKE gene families in barley, rice and Arabidopsis. Plant Physiol 131:1855-1867.
19. Orna BN, Ravit E, Anna P, et al. (2006) The CCAAT binding factor can mediate interactions between CONSTANS-like proteins and DNA. Plant J 46:462-476.
20. Robert LS, Robson F, Sharpe A, et al. (1998) Conserved structure and function of the *Arabidopsis* flowering time gene CONSTANS in *Brassica napus*. Plant Mol Biol 375: 763-772.
21. Holefors A, Opseth L, Rosnes AKR, Ripel L, Snipen L, Fos-sdal CG, and Olsen JE (2009) Identification of PaCOL1 and PaCOL2, two CONSTANS-Like genes showing decreased transcript levels preceding short day induced growth cessation in Norway spruce. Plant Physiology and Biochemistry 47:105-115
22. Borden K L (1998) RING fingers and B-boxes: zinc-binding protein-protein interaction domains. Biochem Cell Biol 762: 351-358.
23. Corbesier L, Coupland G (2005) Photoperiodic flowering of Arabidopsis: integrating genetic and physiological approaches to characterization of the floral stimulus. Plant Cell and Environment 28: 54-66.
24. Strayer C, Oyama T, Schultz TF, et al. (2000) Cloning of the Arabidopsis clock gene TOC1, an autoregulatory response regulator homolog. Science 289: 768-771.
25. Torok M, Elkin LD (2000) Two B or not two B? Overview of the rapidly expanding B-box family of proteins. Differentiation 673: 63-71.
26. Khanna R, Kronmiller B, Maszle DR, et al. (2009) The Arabidopsis B-box zinc finger family.

Plant Cell 21: 3416-3420.

27. Robson F, Costa MM, Hepworth SR, et al. (2001) Functional importance of conserved domains in the flowering-time gene CONSTANS demonstrated by analysis of mutant alleles and transgenic plants. Plant J 28: 619-631.

28. Ben NO, Eshed R, Parnis A, et al. (2006) The CCAAT binding factor can mediate interactions between CONSTANS-like proteins and DNA. Plant J 46: 462-476.

29. Griffiths S, Dunford RP, Coupland G, et al. (2003) The evolution of CONSTANS-like gene families in barley, rice, and *Arabidopsis*. Plant Physiol 1314: 1855-1867.

30. Jang S, Marchal V, Panigrahi KCS, et al. (2008) *Arabidopsis* COP1 shapes the temporal pattern of CO accumulation conferring a photoperiodic flowering response. EMBO J 27: 1277-1288.

31. Kumagai T, Ito S, Nakamichi N, Niwa Y, Murakami M, Yamashino T, Mizuno T (2008) The common function of a novel subfamily of B-box zinc finger proteins with reference to circadian-associated events in Arabidopsis thaliana. Biosci Biotechnol Biochem 72: 1539-1549

32. Indorf M, Cordero J, Neuhaus G, Rodrguez FM (2007) Salt tolerance (STO), a stress-related protein, has a major role in light signaling. Plant J 51: 563~574

33. Datta S, Hettiarachchi C, Johansson H, Holm M (2007) SALT TOLERANCE HOMOLOG2, a B-box protein in Arabidopsis that activates transcription and positively regulates light mediated development. Plant Cell 19: 3242-3255

34. Datta S, Johansson H, Hettiarachchi C, Irigoyen ML, Desai M, Rubio V, Holm M (2008) LZF1/SALT TOLERANCE HOMOLOG3, an Arabidopsis B-box protein involved in light-dependent development and gene expression, undergoes COP1-mediated ubiquitination. Plant Cell 20: 2324-2328

35. Chang CJ, Li YH, Chen LT, Chen WC, Hsieh WP, Shin J, Choi G, Hu JM (2008) LZF1, a HY5-regulated transcriptional factor, functions in Arabidopsis de-etiolation. Plant J 54: 205-219

36. Ral JP, Colleoni C, Wattebled F, Dauville ED, Nempont C. Deschamps P, Li Z, Morell MK, Chibbar R, Purton S, et al. (2006) Circadian Clock Regulation of Starch Metabolism Establishes GBSSI as a Major Contributor to Amylopectin Synthesis in *Chlamydomonas* reinhardtii. Plant Physiol 142:305–317.

37. Harris EH (1989) The Chlamydomonas source book: a comprehensive guide to biology and laboratory use. Academic Press, San Diego, CA.

38. Deng XD, Gu B, Li YJ, Hu XW, Guo JC and Fei XW (2012) The Roles of acyl-CoA: Diacylglycerol Acyltransferase 2 Genes in the Biosynthesis of Triacylglycerols by the Green Algae *Chlamydomonas reinhardtii*. *Mol Plant* 5:945-947.

39. Deng XD, Li YJ, Fei XW (2011) The mRNA abundance of pepc2 gene is negatively correlated with oil content in *Chlamydomonas reinhardtii*. *Biomass Bioenerg* 35: 1811-1817.

40. Liu B, Vieler A, Li C, Daniel Jones A, Benning C. (2013)Triacylglycerol profiling of microalgae Chlamydomonas reinhardtii and Nannochloropsis oceanica. Bioresour Technol. 146:310-316.

41. La Russa M, Bogen C, Uhmeyer A, Doebbe A, Filippone E, Kruse O, Mussgnug J H (2012) Functional analysis of three type-2 DGAT homologue genes for triacylglycerol production in the green microalga Chlamydomonas reinhardtii. Journal of biotechnology, 162:13-20

42. Gao CF, Xiong W, Zhang YL, Yuan WQ, Wu QY (2008) Rapid quantitation of lipid in microalgae by time-domain nuclear magnetic resonace. *J Microbiol Meth* 75:437-440.

43. Huang GH, Chen G, Chen F (2009) Rapid screening method for lipid production in alga based on Nile red fluorescence. *Biomass Bioenerg* 33:1386-1392.

44. Chen W, Zhang C, Song L, Sommerfeld M, Hu Q (2009) A high throughput Nile red method for quantitative measurement of neutral lipids in microalgae. *J Microbiol Meth* 77:41-47.

45. Li YJ, Fei XW and Deng XD (2012) Novel molecular insights into nitrogen starvation-induced triacylglycerols accumulation revealed by differential gene expression analysis in green algae Micractinium pusillum. *Biomass Bioenerg* 42:199-211.

46. Kindle KL (1990) High frequency nuclear transformation of *Chlamydomonas reinhardtii*. *Proc Natl Acad Sci USA* 87, 1228-1232.

47. Fei XW. and Deng XD (2007) A novel Fe deficiency responsive element (FeRE) regulates the expression of atx1 in *Chlamydomonas reinharditii*. *Plant Cell Physiol* 48: 1496-1503.

48. Livak KJ and Schmittgen TD (2001) Analysis of relative gene expression data using real-time quantitative PCR and the 2(-Delta Delta C(T)). *Method Methods* 25: 402-408.

Figure Legends

Figure 1 Growth curve and lipid contents of *C. reinhardtii* CC425 cultivated in LD or SD conditions. A) Growth curve of *C. reinhardtii* CC425 cultivated in LD conditions and at -S or- P limitation conditions; B）Growth curve of *C. reinhardtii* CC425 cultivated in SD conditions and at -S or -P limitation conditions; C) Lipid contents of *C. reinhardtii* CC425 cultivated in LD conditions and at -S or -P limitation conditions; D) Lipid contents of *C. reinhardtii* CC425 cultivated in SD conditions and at -S or -P limitation conditions. HSM, cells cultivated in the HSM medium; HSM-S, cells cultivated in the S-free HSM medium; and HSM-P, cells grown in the P-free HSM medium.

Figure 2 Microscopic observations of *C. reinhardtii* under LD or SD conditions after four days of cultivation. Algal cell was observed using a Zeiss fluorescence microscope (10X25) after staining with Nile Red dye. Orange fluorescence indicates that the oil droplet is mainly composed of TAG. LD conditions (16 h day time and 8 h dark time); SD conditions (8 h day time and 16 h dark time). HSM, cells cultivated in the HSM medium; HSM-S, cells cultivated in the S-free HSM medium; and HSM-P, cells grown in the P-free HSM medium. The scale bar indicated in the figure is 3 μ m.

Figure 3 Abundance of mRNA of *CrCO* in HSM, HSM-N, and HSM-P media. mRNA levels of *C. reinhardtii* CC425 samples grown in the indicated medium for 1, 2, 3, or 4 d in full daylight were analyzed via RT-PCR . HSM, cells cultivated in the HSM medium; HSM-S, cells cultivated in the S-free HSM medium; and HSM-P, cells grown in the P-free HSM medium.

Figure 4 The biomass, lipid content detected by Nile Red fluorescence method and TAG level, and the mRNA abundance of *CrCO* in *CrCO* RNAi transgenic *C. reinhardtii*. Cr.CC425, *C. reinhardtii* CC425; Cr.Maa7, pMaa7IR/XIR transgenic algae strain; CrCO RNAi18 (31, 87), and pMaa7IR/CrCOIR transgenic algae strains. Statistical analysis was performed using SPSS statistical software. Significance is indicated as *P < 0.05, ** P < 0.01.

Figure 5 mRNA abundance of *PDAT3, DGAT2*, *PFK1, FBP1, CIS,* and *PAP2* in *CrCO* RNAi transgenic algae strain, CrCO RNAi18. Cr CC425, *C. reinhardtii* CC425; Cr.Maa7, pMaa7IR/XIR transgenic algae strain; CrCO RNAi18, and pMaa7IR/CrCOIR transgenic algae strain number 18.

Fig 6 Microscopic observations of *CrCO* transgenic *C. reinhardtii.* After six days of cultivation in full daylight and HSM medium, more oil droplets of *CrCO* RNAi transgenic algae were found. Cr.Maa7, pMaa7IR/XIR transgenic algae strain; CrCO RNAi18, and pMaa7IR/CrCOIR transgenic algae strain number 18. The scale bar indicated in the figure is 2 μm.

Figure 7 Lipid contents of *CrCO* transgenic strains cultivated at LD or SD conditions condition. A) Lipid contents of *CrCO* transgenic strains cultivated under SD conditions in the HSM medium; B) Lipid contents of *CrCO* transgenic strains cultivated under LD conditions in the HSM medium; C) Lipid contents of *CrCO* transgenic strains cultivated under SD conditions in the HSM-P medium; D) Lipid contents of *CrCO* transgenic strains cultivated under LD conditions in the HSM-P medium; E) Lipid contents of *CrCO* transgenic strains cultivated under SD conditions in the HSM-S medium; F) Lipid contents of *CrCO* transgenic strains cultivated under LD conditions in the HSM-S medium;. Cr.Maa7, pMaa7IR/XIR transgenic algae strain; CrCO RNAi18 (31, 87), and pMaa7IR/CrCOIR transgenic algae strains.

Figure 8 Biomass and lipid content detected via the Nile Red fluorescence method, TAG level, and mRNA abundance of *CrCO* in *CrCO*-overexpressed transgenic *C. reinhardtii*. Cr.CC425, *C. reinhardtii* CC425; Cr.pCAMBIA, pCAMBIA1302 transgenic algae strain; pCACO11 (64, 91), and pCAMCO transgenic algae strains. Statistical

analysis was performed using SPSS. Significance is indicated as *P < 0.05, ** P < 0.01.

Figure 9 Lipid content in transgenic algae line detected via Nile red staining. After six days of cultivation in the HSM medium, little oil droplets of *CrCO* transgenic algae were found. Cr.pCAMBIA, pMCAMBIA1302 transgenic algae strain; pCACO64, and pCAMCO transgenic algae strain number 64. The scale bar indicated in the figure is 2 μm.

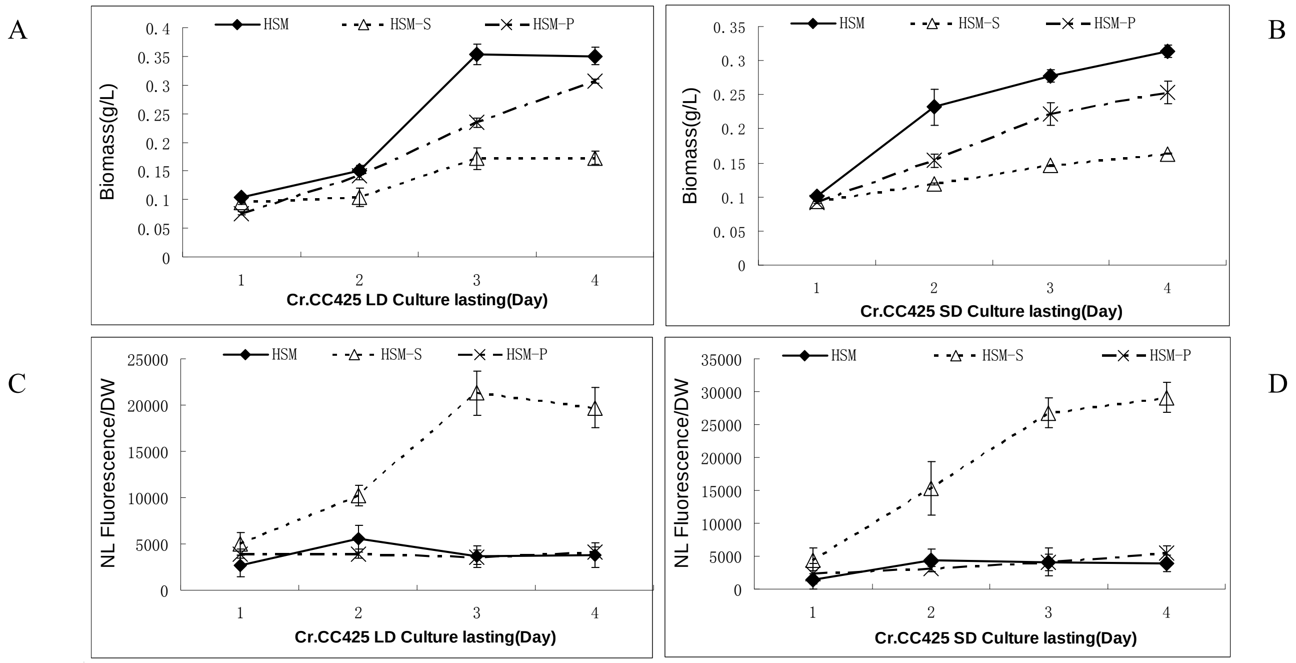
A
B
C
D
HSM
HSM-S
HSM-P
Biomass(g/L)
NL Fluorescence/DW
Cr.CC425 LD Culture lasting(Day)
Cr.CC425 SD Culture lasting(Day)

Figure 1

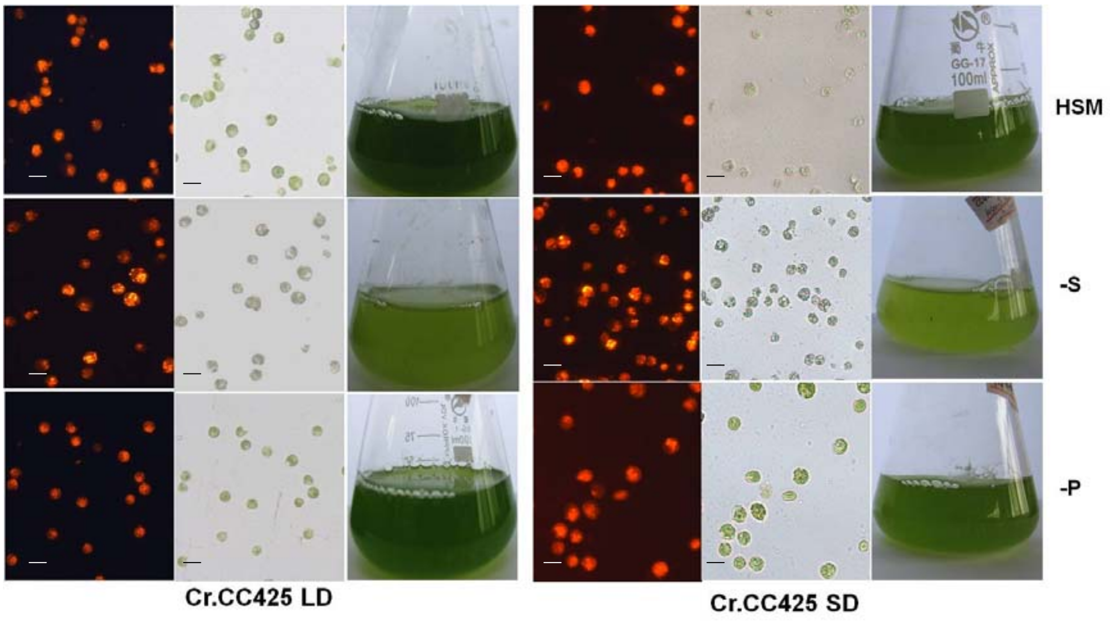

Figure 2

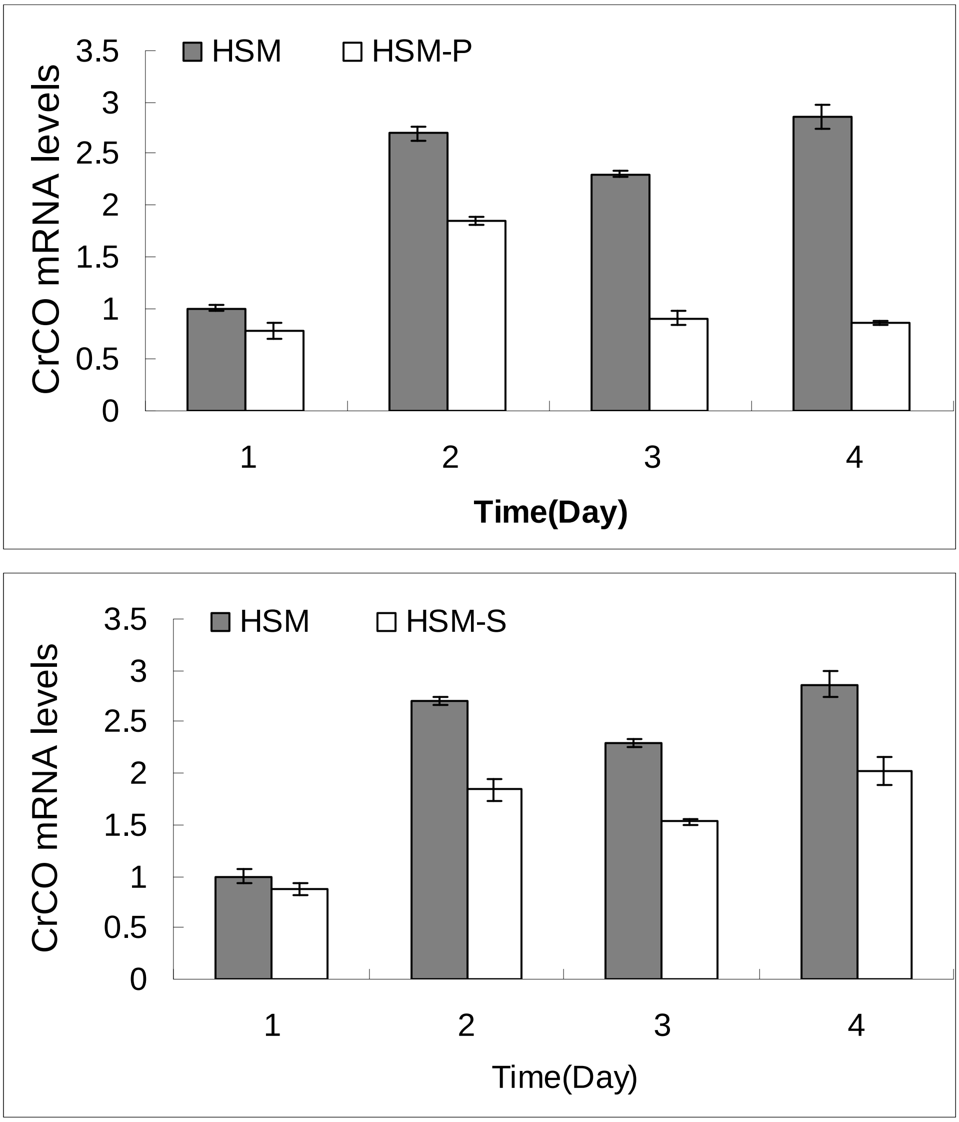
HSM
HSM-P
CrCO mRNA levels
3.5
3
2.5
2
1.5
1
0.5
0
1
2
3
4
Time(Day)
HSM
HSM-S
CrCO mRNA levels
3.5
3
2.5
2
1.5
1
0.5
0
1
2
3
4
Time(Day)

Figure 3

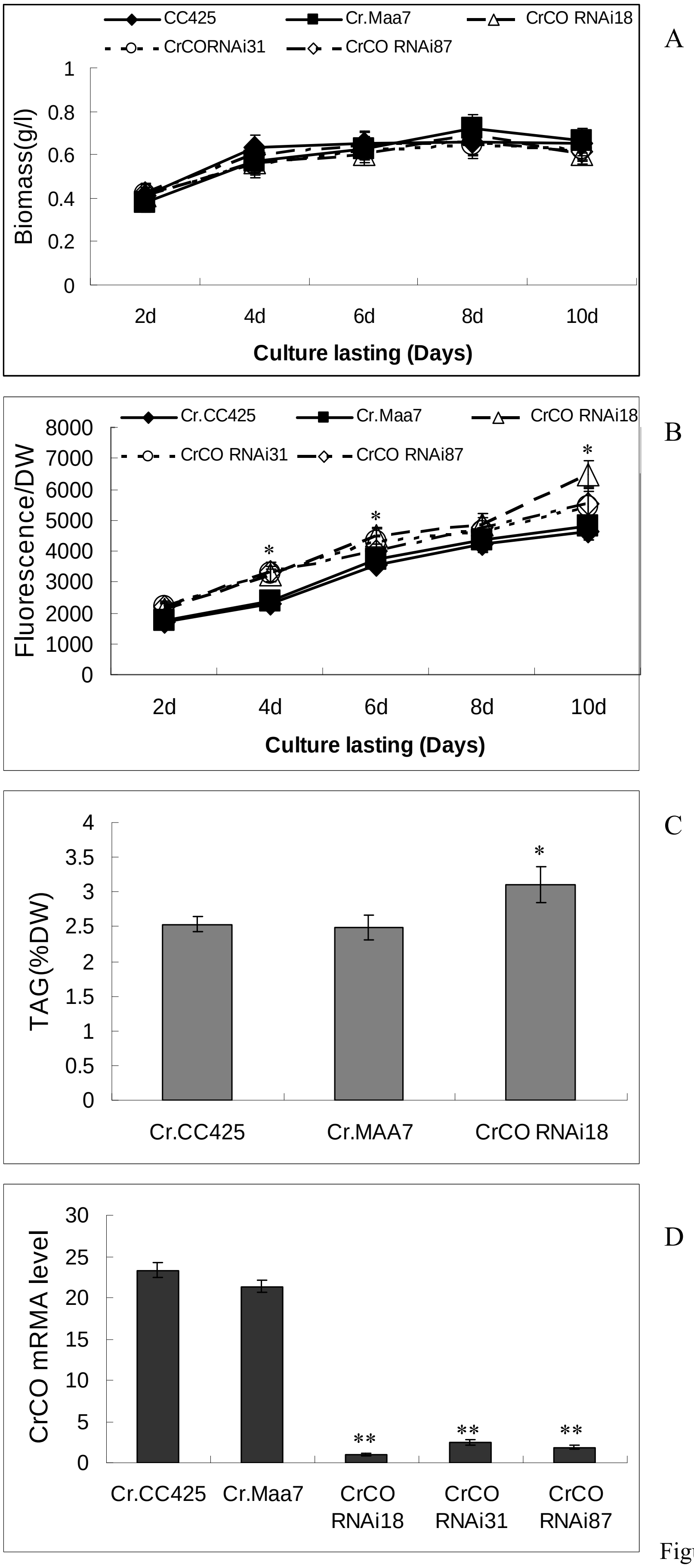

A
CC425
Cr.Maa7
CrCO RNAi18
CrCORNAi31
CrCO RNAi87
Biomass(g/l)
1
0.8
0.6
0.4
0.2
0
2d
4d
6d
8d
10d
Culture lasting (Days)
B
Cr.CC425
Cr.Maa7
CrCO RNAi18
CrCO RNAi31
CrCO RNAi87
Fluorescence/DW
8000
7000
6000
5000
4000
3000
2000
1000
0
*
*
*
2d
4d
6d
8d
10d
Culture lasting (Days)
C
TAG(%DW)
4
3.5
3
2.5
2
1.5
1
0.5
0
*
Cr.CC425
Cr.MAA7
CrCO RNAi18
D
CrCO mRMA level
30
25
20
15
10
5
0
**
**
**
Cr.CC425
Cr.Maa7
CrCO RNAi18
CrCO RNAi31
CrCO RNAi87

Figure 4

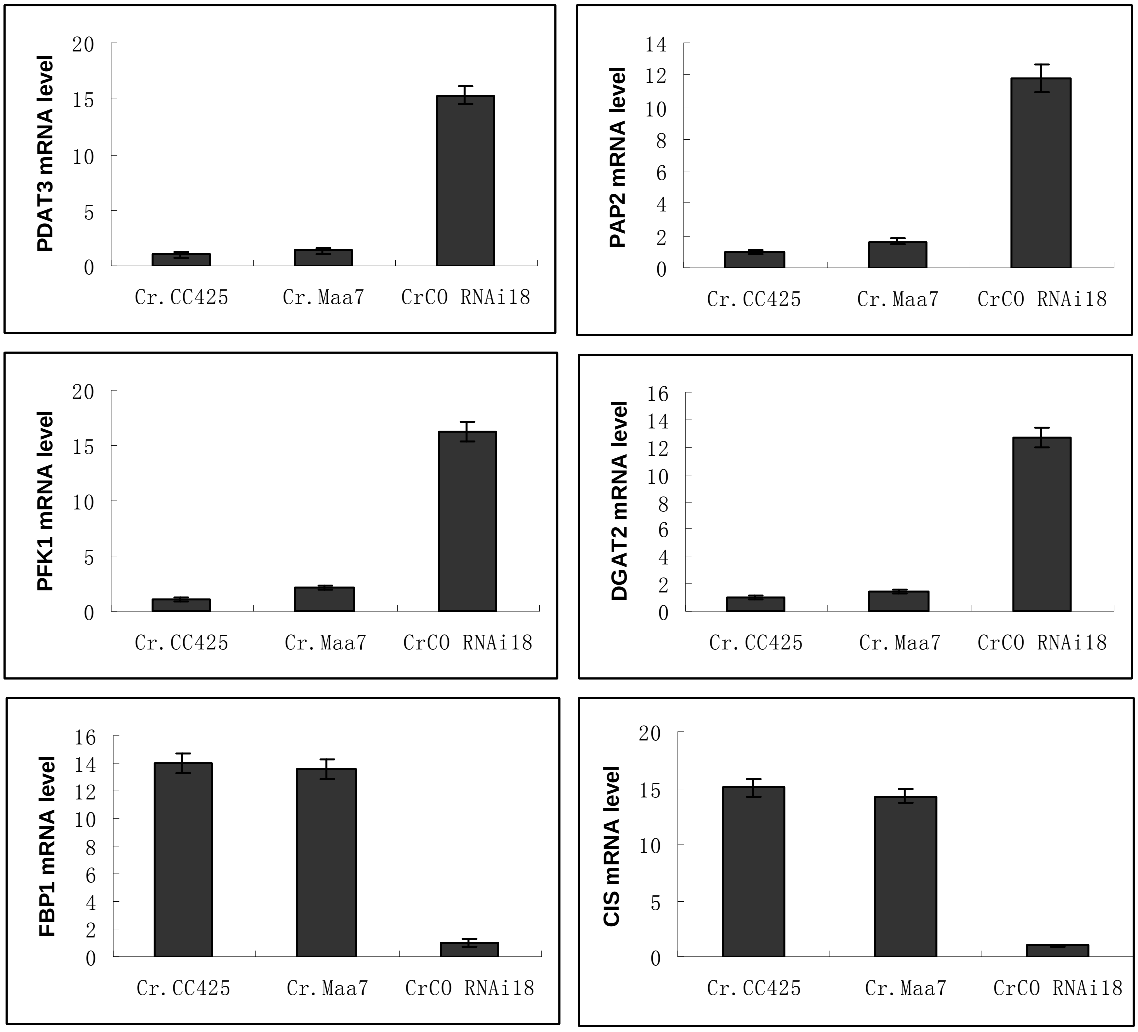
PDAT3 mRNA level
20
15
10
5
0
Cr.CC425
Cr.Maa7
CrCO RNAi18
PAP2 mRNA level
14
12
10
8
6
4
2
0
Cr.CC425
Cr.Maa7
CrCO RNAi18
PFK1 mRNA level
20
15
10
5
0
Cr.CC425
Cr.Maa7
CrCO RNAi18
DGAT2 mRNA level
16
14
12
10
8
6
4
2
0
Cr.CC425
Cr.Maa7
CrCO RNAi18
FBP1 mRNA level
16
14
12
10
8
6
4
2
0
Cr.CC425
Cr.Maa7
CrCO RNAi18
CIS mRNA level
20
15
10
5
0
Cr.CC425
Cr.Maa7
CrCO RNAi18

Figure 5

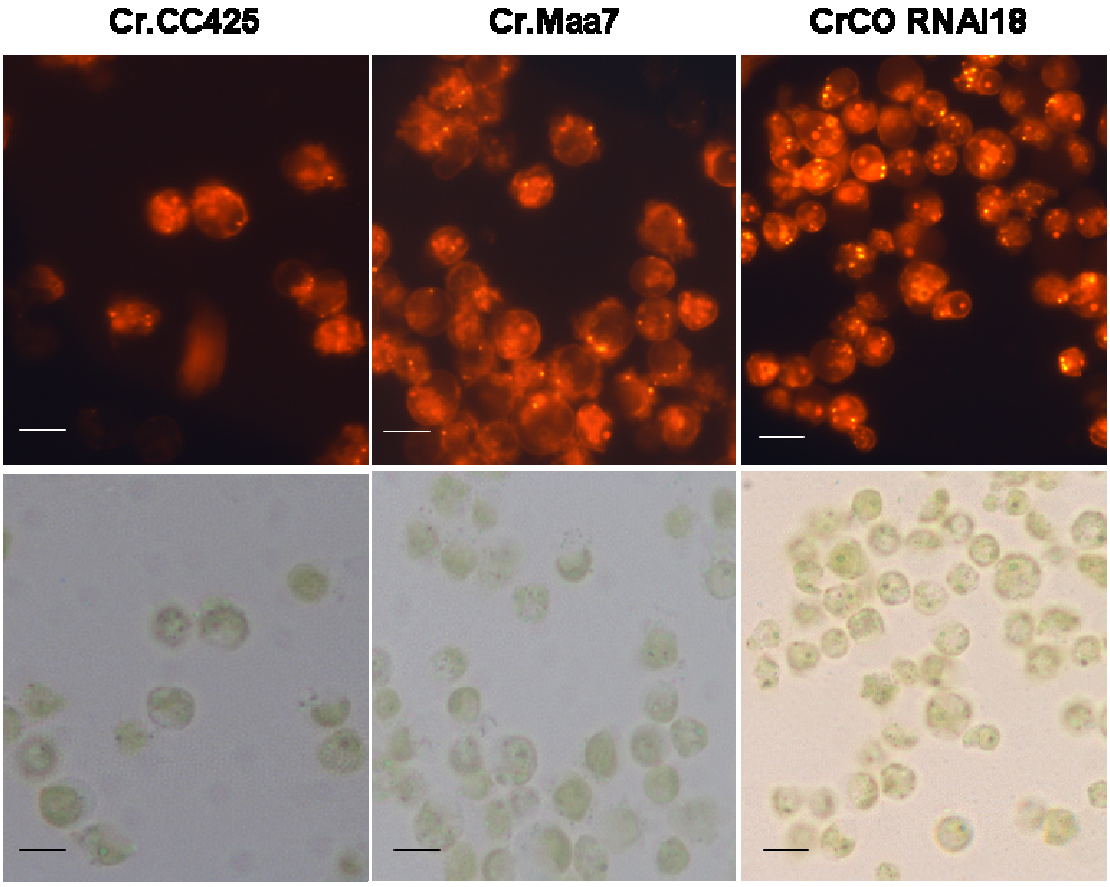

Figure 6

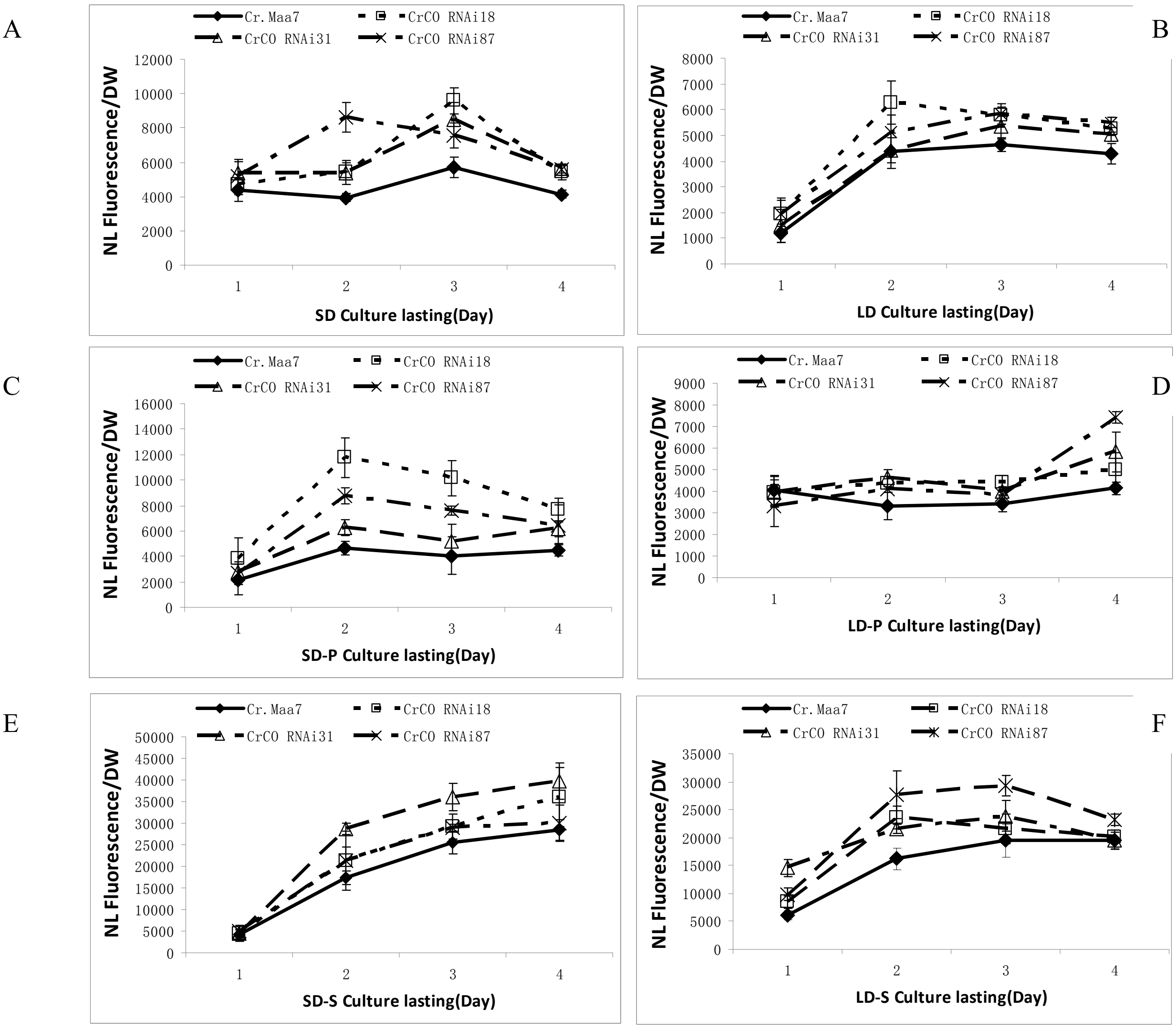
A
B
C
D
E
F
Cr. Maa7
CrCO RNAi18
CrCO RNAi31
CrCO RNAi87
NL Fluorescence/DW
SD Culture lasting(Day)
LD Culture lasting(Day)
SD-P Culture lasting(Day)
LD-P Culture lasting(Day)
SD-S Culture lasting(Day)
LD-S Culture lasting(Day)

Figure 7

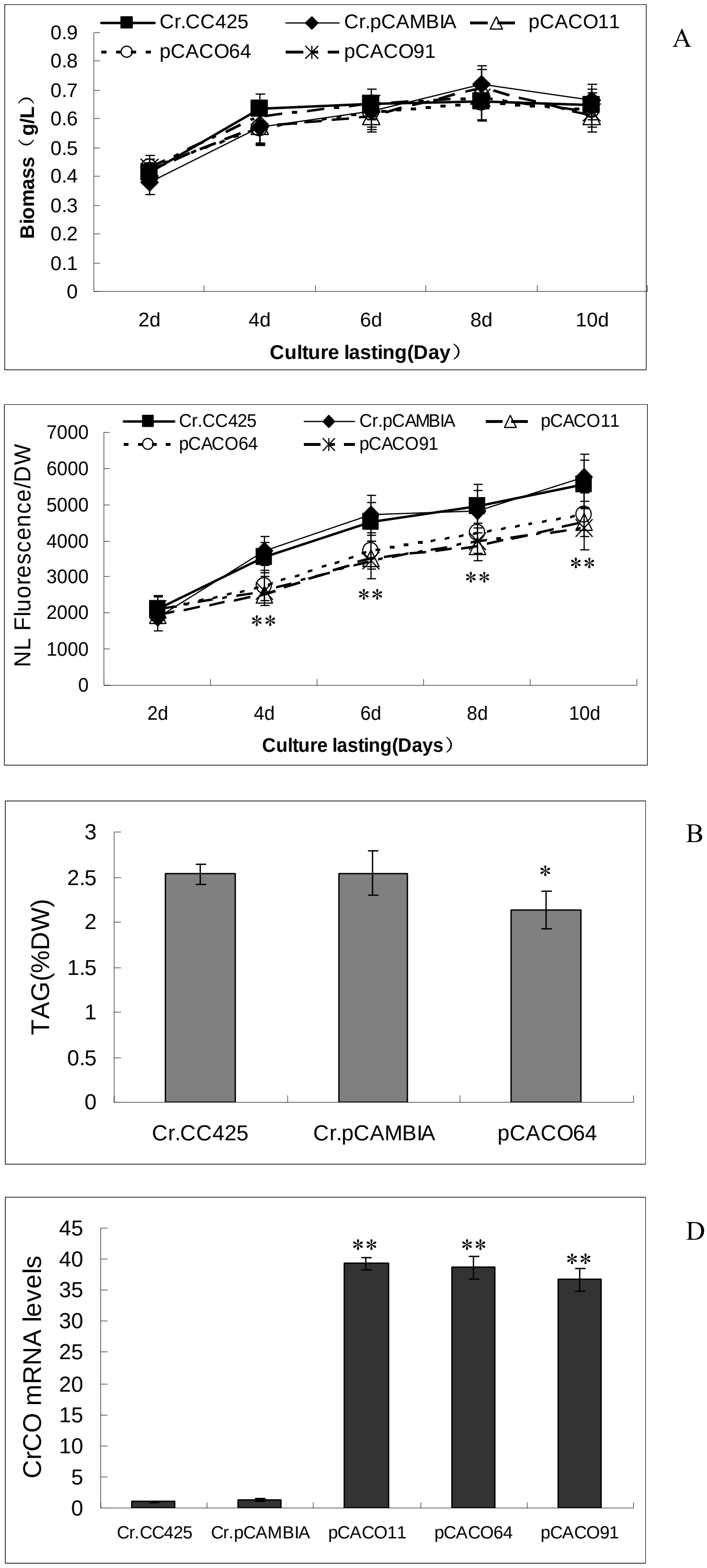
A
Cr.CC425
Cr.pCAMBIA
pCACO11
pCACO64
pCACO91
Biomass（g/L）
0.9
0.8
0.7
0.6
0.5
0.4
0.3
0.2
0.1
0
2d
4d
6d
8d
10d
Culture lasting(Day）
Cr.CC425
Cr.pCAMBIA
pCACO11
pCACO64
pCACO91
NL Fluorescence/DW
7000
6000
5000
4000
3000
2000
1000
0
**
**
**
**
2d
4d
6d
8d
10d
Culture lasting(Days）
B
TAG(%DW)
3
2.5
2
1.5
1
0.5
0
*
Cr.CC425
Cr.pCAMBIA
pCACO64
D
CrCO mRNA levels
45
40
35
30
25
20
15
10
5
0
**
**
**
Cr.CC425
Cr.pCAMBIA
pCACO11
pCACO64
pCACO91

Figure 8

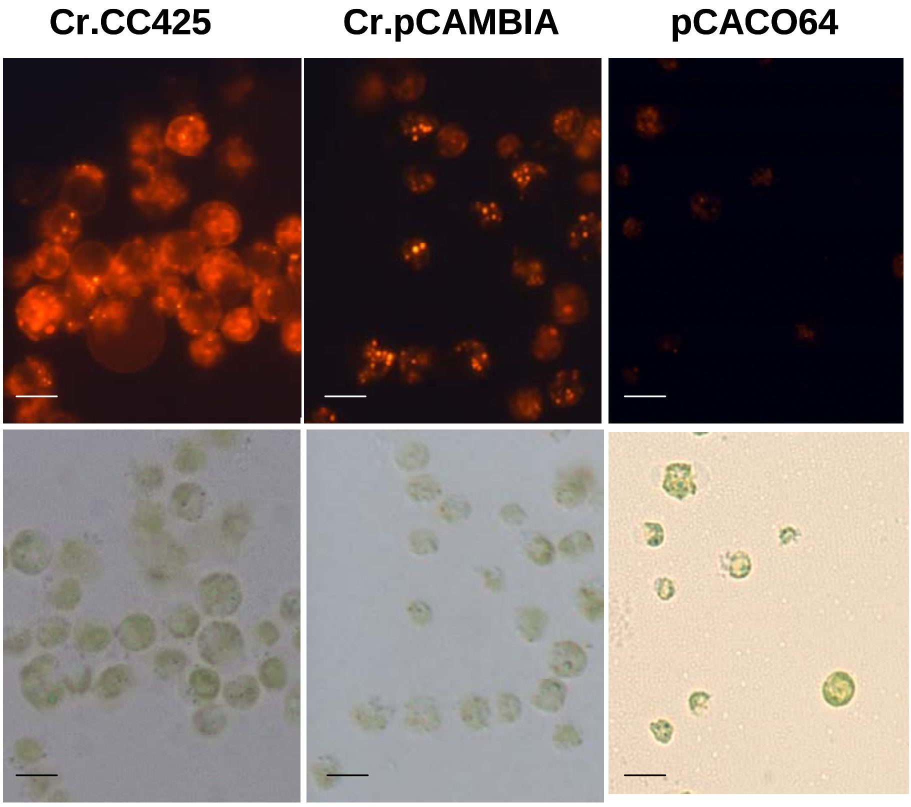

Figure 9
